# Isolated external iliac artery aneurysm: a rare case presentation of IgG4-related disease

**DOI:** 10.1590/1677-5449.202201192

**Published:** 2023-04-07

**Authors:** Naveen Maheshwari, Venkata Vineeth Vaddavalli, Kishore Abuji, Ajay Savlania, Ritambhra Nada

**Affiliations:** 1 Post Graduate Institute of Medical Education and Research - PGIMER, Chandigarh, India.

**Keywords:** iliac artery aneurysm, vasculitis, IgG4-related disease, iliofemoral bypass, aneurisma de artéria ilíaca, vasculite, doença relacionada à IgG4, bypass iliofemoral

## Abstract

Isolated external iliac artery aneurysm is a rare occurrence. These aneurysms have varied presentations depending on size and proximity. Both open surgical and endovascular modalities can be used for treatment depending upon presentation, aneurysmal anatomy, and patient condition. Preservation of at least one internal iliac artery is important to prevent post-repair hypogastric ischemia. There are no previous reports of IgG4-related disease (IgG4-RD) as etiology of these aneurysms. A 32-year-old male patient presented with a left lower abdominal lump and was found to have a left external iliac artery aneurysm on computed tomography angiography. The patient underwent iliofemoral bypass with an 8 mm polyester graft. Histopathological examination of the aneurysm wall suggested IgG4-RD. The patient fulfilled the 2020 Revised Comprehensive Diagnostic Criteria for IgG4-RD. An 18-Fluorodeoxyglucose-Positron Emission Tomography scan performed in the postoperative period showed no active disease, hence medical therapy was not instituted. The patient is doing well at 1 year.

## Resumo

O aneurisma isolado da artéria ilíaca externa é uma ocorrência rara. Esses aneurismas têm apresentações variadas, dependendo do tamanho e da proximidade. Ambas as modalidades cirúrgicas aberta e endovascular podem ser usadas para o tratamento, dependendo da apresentação, anatomia do aneurisma e condição do paciente. A preservação de pelo menos uma artéria ilíaca interna é importante para prevenir isquemia hipogástrica pós-reparação. A doença relacionada à imunoglobulina G4 (IgG4-RD) nunca havia sido encontrada como etiologia desse aneurisma. Um paciente do sexo masculino de 32 anos que apresentava um nódulo no abdome inferior esquerdo foi diagnosticado com aneurisma da artéria ilíaca externa esquerda na angiotomografia computadorizada. O paciente foi submetido a *bypass* iliofemoral com enxerto de poliéster de 8 mm. O exame histopatológico da parede do aneurisma era indicativo de IgG4-RD. O paciente cumpriu os Critérios Abrangentes Revisados ​​para IgG4-RD de 2020. A tomografia por emissão de pósitrons com 18-fluorodesoxiglicose no pós-operatório não evidenciou doença ativa, não sendo instituída terapia medicamentosa. Após seguimento de 1 ano, o paciente está bem.

## INTRODUCTION

Isolated external iliac artery aneurysms (EIAA) are rare and seldom reported. Isolated iliac artery aneurysms (IAA) comprise less than 2% of all intra-abdominal aneurysms. In turn, isolated EIAAs account for less than 10% of isolated IAAs. The exact incidence and gender predilection of isolated EIAAs are unknown.^1^ They have been described in patients with ages ranging from 27 to 78 years.^2^^,^^3^ Although most of the reported cases were unilateral, bilateral isolated EIAAs have also been reported.^4^ The presentation of isolated EIAA can vary, ranging from asymptomatic to hemorrhagic shock in an emergency. With a natural resistance to aneurysm formation, EIAAs have shown diverse etiologies, but IgG-4-related disease (IgG4-RD) has never been found to cause isolated EIAA. Here, we report a case of a young male who presented with a painful pulsatile lump in the left iliac fossa, which on evaluation was found to be a left EIAA. It was managed surgically by iliofemoral bypass and final histopathology showed that its etiology was IgG4-related aortoarteritis.

This case report complies with the Helsinki Declaration and Indian Council of Medical Research guidelines. Written and informed consent was obtained from the patient and approval was granted by the institutional ethics committee at the Postgraduate Institute of Medical Education and Research, Chandigarh, India (Ref. no. INT/IEC/2022/00968).

## CASE REPORT

**Figure 1:**
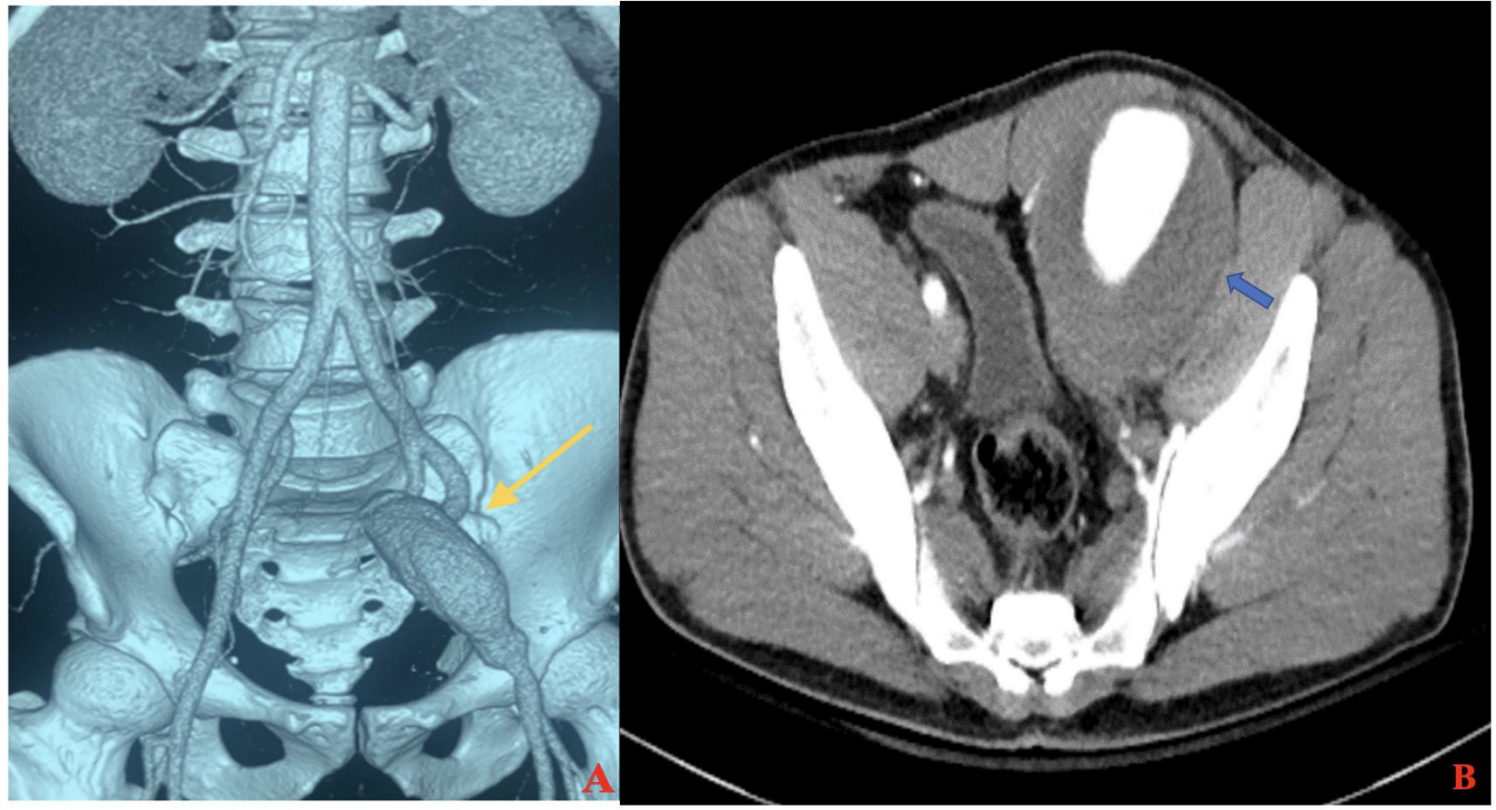
Computed tomography - (A) Volume rendered image- anteroposterior view showing left external iliac artery aneurysm (yellow arrow); (B) cross-sectional image showing aneurysmal dilation of left external iliac artery with intramural thrombus (blue arrow). The right external iliac artery and bilateral internal iliac arteries are normal.

A 32-year-old male, chronic smoker with no comorbidities presented with a lump and dull aching pain in the left iliac fossa, with duration of 12 months and 8 months, respectively. The lump was pulsatile, gradually increased in size, and was 6X6 cm at presentation. The patient complained of occasional heaviness in the left lower limb associated with swelling, which decreased on lying down. Previously, there was no history of trauma or claudication in the left lower limb. On abdominal examination, a 10X6 cm pulsatile vague lump with dilated overlying veins was palpable in the left lower quadrant. Pitting edema with pigmentation was present in the left leg without any dilated veins. All peripheral pulses were palpable. Computed Tomography Angiography (CTA) of the abdomen and bilateral lower limbs showed focal fusiform dilatation of the left external iliac artery (6X6.3X8.7 cm), 4.6 cm distal to the bifurcation of the left common iliac artery, with eccentric thrombus, and a proximal neck of 10.7 mm and distal outlet of 10.7 mm. The venous system was unremarkable (Figure 1). In inflammatory blood workup, LDH and CRP were normal. Tests for cANCA, pANCA, and ANA were negative.

**Figure 2:**
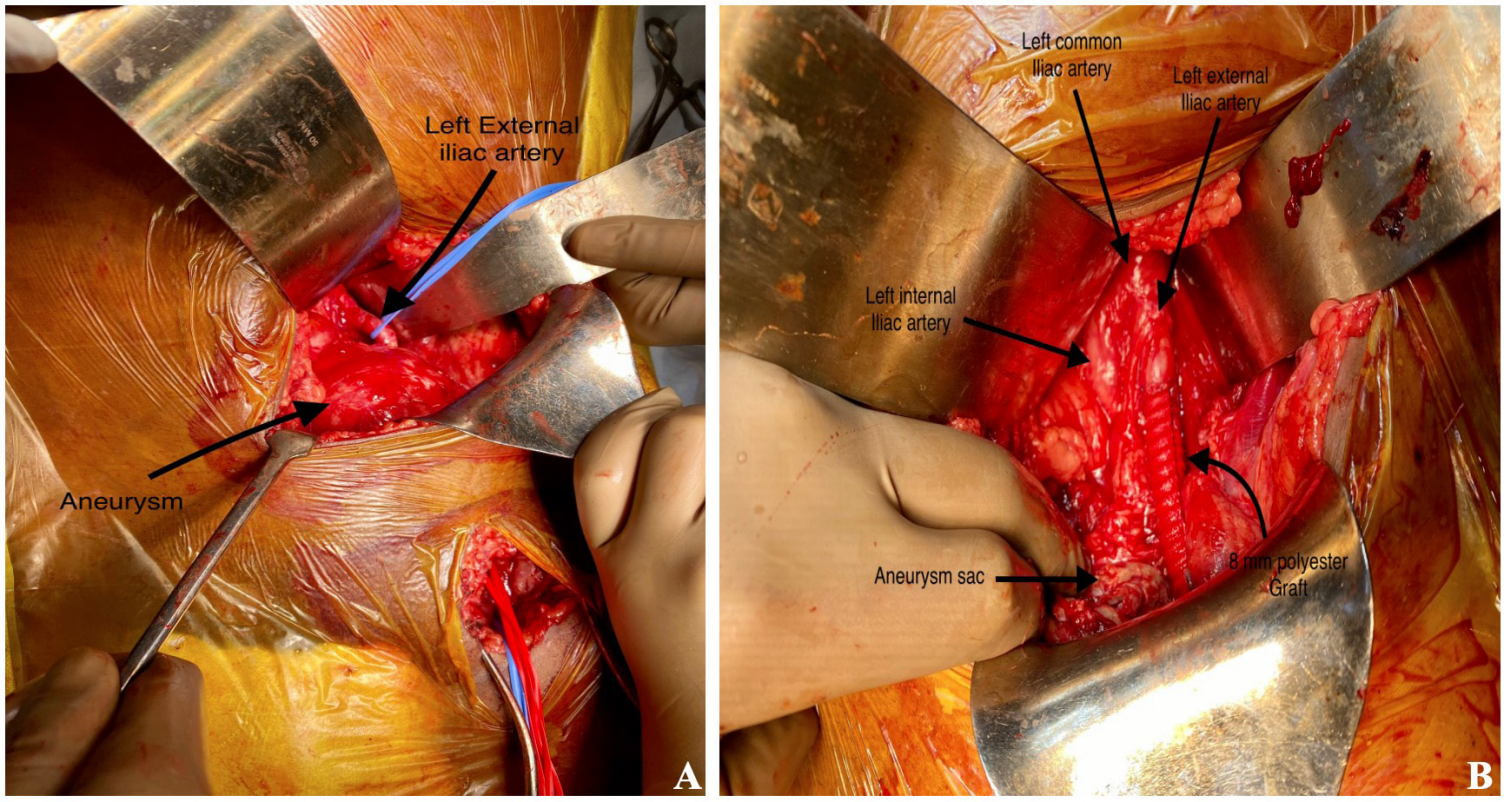
Intra-operative images (A) Large left external iliac artery aneurysm with proximal (blue silastic tape looping left EIA) and distal (red silastic tape looping left CFA) vascular control with Silastic tapes; (B) Reconstruction with 8mm polyester graft.

After discussion with the patient, he was scheduled for open surgical repair because of his young age and preserved physiology. Intraoperatively, the retroperitoneal approach was used. The left femoral artery was exposed from a different incision in the groin. Proximal and distal controls were taken at the left external iliac artery and the left common femoral artery. Dense adhesions were present adjacent to the aneurysm. Nearly 250 ml of clots were evacuated upon opening the sac. The aneurysmal wall was thicker in comparison to the proximal and distal normal arterial walls. Iliofemoral bypass was done using an 8 mm polyester graft from the left external iliac artery to the left common femoral artery (Figure 2). There was no difficulty in suturing the graft to the proximal and distal ends. Postoperatively, all distal pulses were palpable, and the course was uneventful.

**Figure 3:**
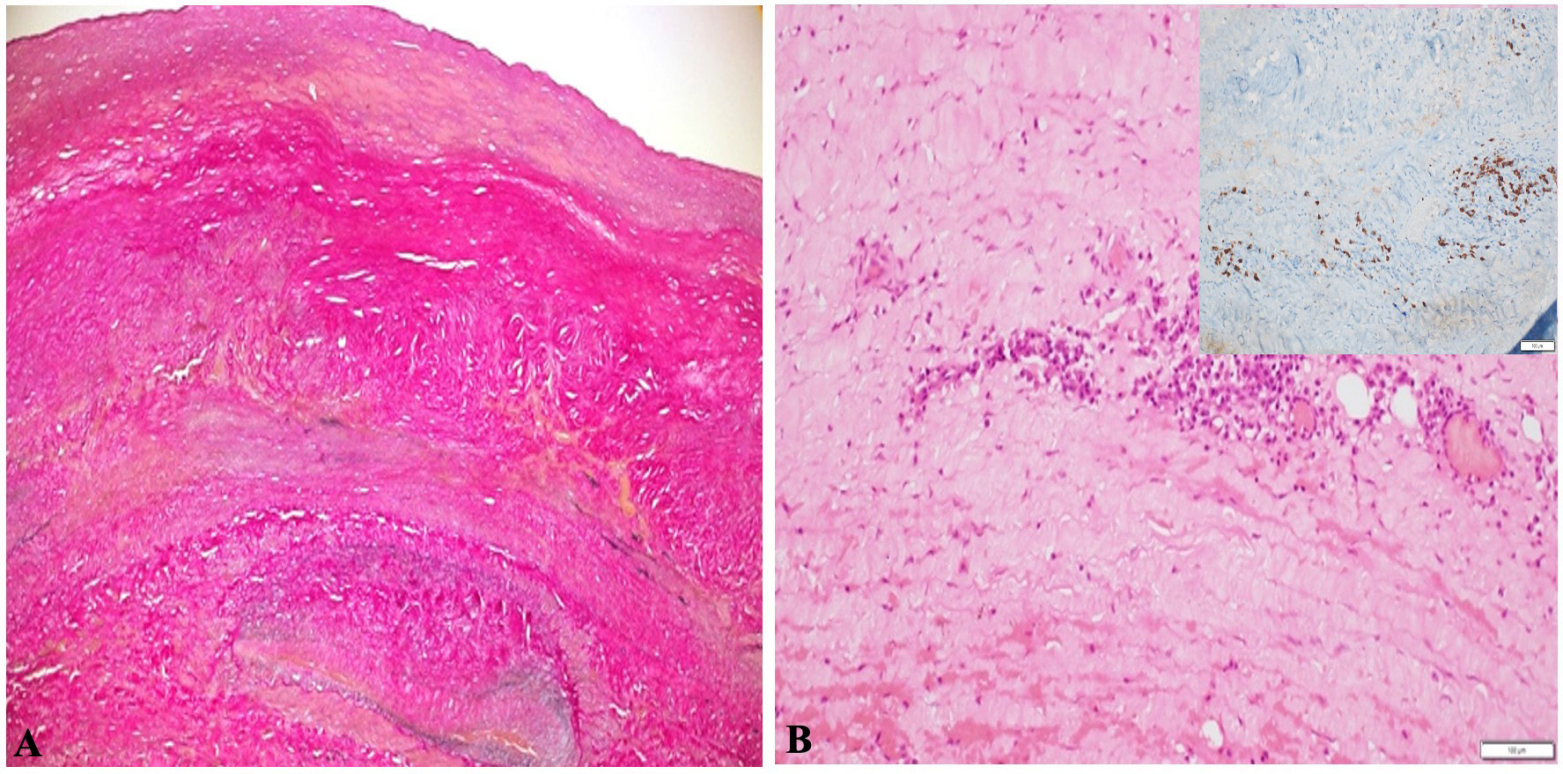
(A) Thickened arterial wall with intimal and adventitial thickening and complete loss of elastic tissue from media; (B) perivascular plasma cell-rich inflammation, which had abundant IgG4-positive cells (inset).

On histopathological examination, the aneurysmal wall showed destruction of media with periadventitial plasma cells and lymphoid infiltrates, which were IgG4 positive. There was evidence of venulitis. There were no atherosclerotic changes in the intima (Figure 3). IgG4 levels were tested and found to be elevated (625 mg/dl) and an 18-Fluorodeoxyglucose-Positron Emission Tomography (FDG-PET) scan was performed to assess disease activity elsewhere in the body. It showed no active uptake except at the operative site, suggestive of postoperative inflammatory changes. At present, after 1 year, the patient is healthy and in follow-up with no signs of a pulsatile lump at the operative site and all peripheral pulses are palpable. Unfortunately, we do not have postoperative imaging for the patient, which is a limitation of this report.

## DISCUSSION

Isolated EIAA is a rare occurrence. There are fewer than a hundred case reports in the available medical literature. Several theories have been proposed to try to explain the rarity of EIAA. The Tilson et al.^5^ developmental etiology theory states that resistance of EIA to aneurysm formation is due to the distinct embryonic lineage of its vascular smooth muscle cells. EIA is considered aneurysmal if the diameter is >18.5 mm in males and >15 mm in females.^6^ The exact etiology of EIAA is unknown. Atherosclerosis has been cited as the most common etiology, as seen elsewhere in the body. Four of the available case reports described cystic medial necrosis as etiology.^7^ In one case report, hyperhomocysteinemia was found to be present.^8^ Other etiologies include trauma, iatrogenic injury, arteritis, connective tissue disorders, and infections.^9^

The aneurysmal wall histological examination showed IgG4-positive plasmacytes in the index case. The IgG4-RD was suspected and, on evaluation, fulfilled all the ‘2020 revised comprehensive diagnostic criteria’^10^- organ involvement, serum IgG4 levels >135 mg/dl, fibrosis and IgG4 positive plasma cells >10/high power field. IgG4-RD has been described as causing primary inflammatory vasculitis and periaortitis (secondary to retroperitoneal fibrosis) in 22.5% of all patients with IgG-RD. However, the distinction between the two forms is not very clear.^11^ Although first attributed to inflammatory abdominal aortic aneurysms (AAA), IgG4-RD typically involves coronary arteries, first and second-order aortic branches.^12^ Of all patients affected with IgG4-RD with vascular involvement, only 36% were shown to develop arterial aneurysms, all with primary inflammatory vasculitis. Other arterial lesions associated with IgG4-RD are arterial stenosis and dissection involving the pulmonary artery, thoracic and abdominal aorta, and coronary, carotid, renal, and iliac arteries. Superior vena cava compression due to periaortitis has also been reported. Obliterative arteritis and phlebitis are found on microscopy.^11^ No case reports exist in the medical literature describing an EIAA associated with IgG4-RD. Kasashima et al.^13^ reported IgG4-RD as the cause of 5% of AAA.

The presentation of isolated EIAA can be asymptomatic or with a pulsatile lump in the lower abdomen, hip pain, groin swelling, vaginal bleeding, intermittent claudication, acute limb ischemia, or hemorrhagic shock. The clinical presentation of EIAA corresponds to its size and proximity to other structures.^4^ IAA of size >6cm are usually symptomatic and have an increased risk of rupture.^14^ The course and prognosis of IAA have been correlated to the rate of expansion. For aneurysms <3 cm, diameter increased at the rate of 1 mm/year, whereas >3 cm diameter aneurysms expanded at 26 mm/year, which increased to 32 mm/year in hypertensive patients.^15^ Smoking cessation has been proposed to reduce the IAA expansion rate, as with AAA.^16^

CTA is the preferred noninvasive modality over conventional angiography for establishing the diagnosis. FDG-PET is an effective noninvasive modality for detecting disease activity and treatment response for various vasculitis syndromes. The sensitivity of FDG-PET for vasculitis is 77 to 92%, whereas specificity is 89 to 100%.^17^

IgG4-RD patients with active disease are managed medically with corticosteroids. Rituximab is effective for resistant and relapsing cases. Presence of symptomatic stenosis or aneurysm is an indication for surgical intervention.^18^ While there is no recommendation for isolated EIAA management, asymptomatic patients with a 3.5 cm IAA diameter are ideal candidates for elective repair. Any symptomatic IAA is an indication for repair, irrespective of diameter.^19^

Endovascular aneurysm repair is the treatment of choice in patients with suitable aneurysmal anatomy and severe cardiopulmonary disease. Open repair of IAA is considered more durable and preferred in young patients, emergencies with rupture, and aneurysms with unsuitable anatomy for endovascular repair.^1^ Hypogastric ischemia is a known complication with either of these techniques and preservation of at least one internal iliac artery is recommended, especially in patients with atherosclerosis. Patients with rich femoral and lumbar collaterals may tolerate hypogastric ischemia, but this cannot always be anticipated. Bilateral IIA occlusion may result in sigmoid damage, spinal cord ischemia, hip or buttock claudication or necrosis, sexual erectile dysfunction, or testicular infarction.^20^ Patients managed conservatively should be followed with ultrasonography at six-monthly intervals. If IAA remains stable over several exams, follow-up can be taken annually.^1^

## CONCLUSION

Aneurysms at uncommon sites such as the external iliac artery warrant consideration of vasculitis due to IgG4-RD as possible etiology while evaluating and planning treatment. An EIAA due to IgG4-RD can be managed with open or endovascular approaches on the same principles with additional evaluation of IgG4-RD disease activity and its medical management.
